# Core‐Shell: Resolving the Dilemma of Hard Carbon Anodes by Sealing Nanoporous Particles With Semi‐Permeable Coatings

**DOI:** 10.1002/anie.202519457

**Published:** 2026-01-22

**Authors:** Paul Alexander Appel, Carsten Prinz, Jian Liang Low, Nahom Enkubahri Asres, Shu‐Han Wu, Annica Freytag, Jonas Krug von Nidda, Nader Amadeu de Sousa, Tim‐Patrick Fellinger

**Affiliations:** ^1^ Division 3.6 Electrochemical Energy Materials Bundesanstalt für Materialforschung und ‐prüfung (BAM) Unter den Eichen 87 12203 Berlin Germany; ^2^ Division 6.3 Structure Analysis Bundesanstalt für Materialforschung und ‐prüfung (BAM) Unter den Eichen 87 12203 Berlin Germany; ^3^ Institute of Chemistry and Biochemistry Freie Universität Berlin Arnimallee 22 14195 Berlin Germany; ^4^ Research Group Operando Battery Analysis Helmholtz‐Zentrum Berlin für Materialien und Energie Hahn‐Meitner‐Platz 1 Berlin 14109 Germany

**Keywords:** Core‐Shell, Hard Carbon Anode, Sodium‐Ion Battery, Diethyl Carbonate Vapor Sorption, Activated Carbon

## Abstract

A core‐shell strategy is introduced to overcome the dilemma of common non‐graphitic hard carbon anodes, linking high reversible storage capacity to practically unacceptable irreversible losses in the first cycle(s). Just as graphite homogeneously combines effective lithium storage with an electrolyte solvent‐sieving function, we show that both of these functions could be strategically integrated into non‐graphitic carbons in a heterogeneous structure. Highly porous activated carbons are sealed by kinetically tuned gas‐phase deposition of non‐graphitic carbon to form a functional core‐shell structure. Gas sorption porosimetry on core, shell, core–shell, and cracked core‐shell particles confirms preserved core porosity and a semi‐permeable shell. Diethyl carbonate sorption analysis is introduced as a more suitable probe than N_2_ or CO_2_ sorption, linking first‐cycle losses to the liquid–solid interface of carbon anodes. The functional core‐shell particles with much reduced diethyl carbonate uptake allow for high storage capacity and reduced first cycle losses. Delivering 400 ± 24 mAh g^−^^1^ with 82 ± 2% first‐cycle reversibility, it is shown that three‐stage Na storage in designed core‐shell anodes can compensate for the larger size of sodium compared to lithium stored in graphite anodes (372 mAh g^−1^). The designed core‐shell anodes show state‐of‐the‐art performance with commercial promise.

## Graphical abstract

A core–shell strategy is presented to overcome the high first‐cycle losses of non‐graphitic hard carbon anodes. Activated carbon is sealed by gas‐phase carbon deposition to create semi‐permeable shells that spatially separate SEI formation from Na storage in a retained nanoporous core. The resulting anode delivers 400 ± 24 mAh g^−1^ with an ICE of 82 ± 2%. Diethyl carbonate vapor sorption quantifies the solid‐liquid interface to predict first‐cycle efficiency for adsorption‐based Na storage.

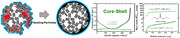

Sodium‐ion batteries (SIBs) have been investigated since the 1970s.^[^ ^1^ ^]^ Following the success of graphite anodes in lithium‐ion batteries (LIBs) with their high energy density and improved safety, interest in SIBs declined.^[^ ^2^, ^3^, ^4^ ^]^ However, the rapid growth in LIB demand and concerns over lithium supply have revived research into SIBs.^[^ ^4^, ^5^ ^]^ They offer clear advantages in the abundance and distribution of raw materials and are a *drop‐in technology*.^[^ ^5^ ^]^ CATL announced market‐ready SIBs in 2021, JAC Group launched the first SIB‐based passenger car (Sehol E10X) in December 2023,^[^ ^1^, ^8^ ^]^ and HiNa Battery introduced a 100 MWh storage system in June 2024^[^ ^6^, ^7^, ^8^, ^9^ ^]^ As sodium ions cannot intercalate into graphite, non‐graphitic carbons such as hard carbons are regarded as the most promising anodes for SIBs. Their larger interlayer spacing (d ≥ 0.4 nm) enables sodium insertion.^[^ ^10^ ^]^ There has been significant discussion in the literature on the sodium storage mechanism. Initially Dahn et al. proposed an intercalation filling model.^[^ ^2^ ^]^ Ji et al. then later proposed the three‐stage mechanism, in which surface adsorption is another mode of storage.^[^ ^11^ ^]^ Although the assignment of features in sodiation/desodiation curves remains controversial, three storage modes are generally accepted: adsorption at edges and defects, intercalation into graphitic‐like nanodomains, and sodium cluster formation.^[^ ^12^ ^]^

Besides the lower density of non‐graphitic carbons, which reduces volumetric energy density, a critical drawback is their low initial Coulomb efficiency (ICE). Most carbons with capacities above 250 mAh g^−^^1^ show poor ICE (Figure S1).^[^ ^13^, ^14^, ^15^, ^16^ ^]^ It is a true dilemma, since _ as a rule of thumb _ high reversible capacities typically go along with small ICEs (Figure S1). For graphite, first‐cycle losses scale with surface area, but this relation is unclear for hard carbons, where structural complexity obscures the trend.^[^ ^10^, ^17^, ^18^ ^]^ Non‐graphitic carbons with high‐capacities (≈550 mAh g^−^^1^) have been reported, but they suffer impractically large first‐cycle losses, sometimes exceeding subsequent reversible capacity (Figure S1).^[^ ^10^ ^]^ Moreover, first cycle capacity losses are often not reported in publications on high capacity carbon anodes.

Thermal annealing has been shown to reduce first‐cycle losses in both Li‐ and Na‐ion batteries by “closing” pores, but this also reduces reversible capacity, as described by the “falling house of cards” model.^[^ ^2^, ^16^ ^]^ Therefore, hard carbons are considered most promising and are found in currently available commercial Na‐ion batteries.^[^ ^4^, ^5^ ^]^ These disordered carbons are typically synthesized by carbonization of biomass at 1300–1600 °C. During annealing, pore closure lowers specific surface area (SSA) and shifts charge–discharge curves from sloping to plateau‐like. This evolution is attributed to increased graphitization, with sloping capacity linked to defects and edges, and the plateau region associated with sodium cluster formation in nanoscopic pores.^[^ ^13^ ^]^

The reduction of irreversible losses in hard carbons has been attributed to “closed” pores, based on their inaccessibility to nitrogen molecules in conventional N_2_ sorption porosimetry. More recently, these pores are described as “molecular sieving” pores, which block electrolyte solvent molecules but remain permeable to sodium ions.^[^ ^19^ ^]^ Consequently, the current research aims at 1) larger volume fractions of closed pores and 2) on the increase of pore diameters with optimized pore entrances (for bottle‐neck pores).^[^ ^19^, ^20^ ^]^ Achieving higher energy density in SIBs requires high reversible anode capacity. The present benchmark is 478 mAh g^−^^1^ with 88% ICE, reported by Komaba et al. for MgO‐templated hard carbons after annealing based on a multi‐step synthetic procedure.^[^ ^21^, ^22^ ^]^ Recently, several groups have explored bottom‐up pore design instead of top‐down annealing. Using chemical vapor deposition (CVD), Cao et al. and Yang et al. introduced closed and molecular‐sieving pores by partially filling existing porosity and narrowing pore entrances, achieving reversible capacities of 348 and 328 mAh g^−^^1^ with ICEs of 82% and 77%, respectively.^[^ ^19^, ^20^ ^]^ In a similar approach, aiming to fill micropores of xerogels, Job et al. obtained a moderate reversible capacity of 298 mAh g^−1^ with a high ICE of 84%.^[^ ^23^ ^]^

In this work we introduce a core–shell strategy for non‐graphitic carbon anodes, where a porous core is sealed with a semi‐permeable shell. This design delivers high reversible capacity while greatly reducing first‐cycle losses. Our aim is to shift the paradigm for understanding reversible capacity and initial losses in non‐graphitic carbon anodes. Instead of focusing solely on pore structure in a homogeneous material, we exploit tailor‐made carbons to spatially separate the two key anode functions in a heterogeneous architecture: the shell provides electrolyte exclusion, while the core serves as the storage medium. In graphite, both functions are realized homogeneously (Scheme 1), but limited to Li storage. As solvent molecules cannot penetrate the particle, the passivating solid–electrolyte interphase (SEI) forms distinctly on the surface, and bulk and surface share the same structure. We show that realizing these functions heterogeneously in non‐graphitic carbons is advantageous and may pave the way to carbon anodes approaching theoretical capacity limits.

**Scheme 1:**
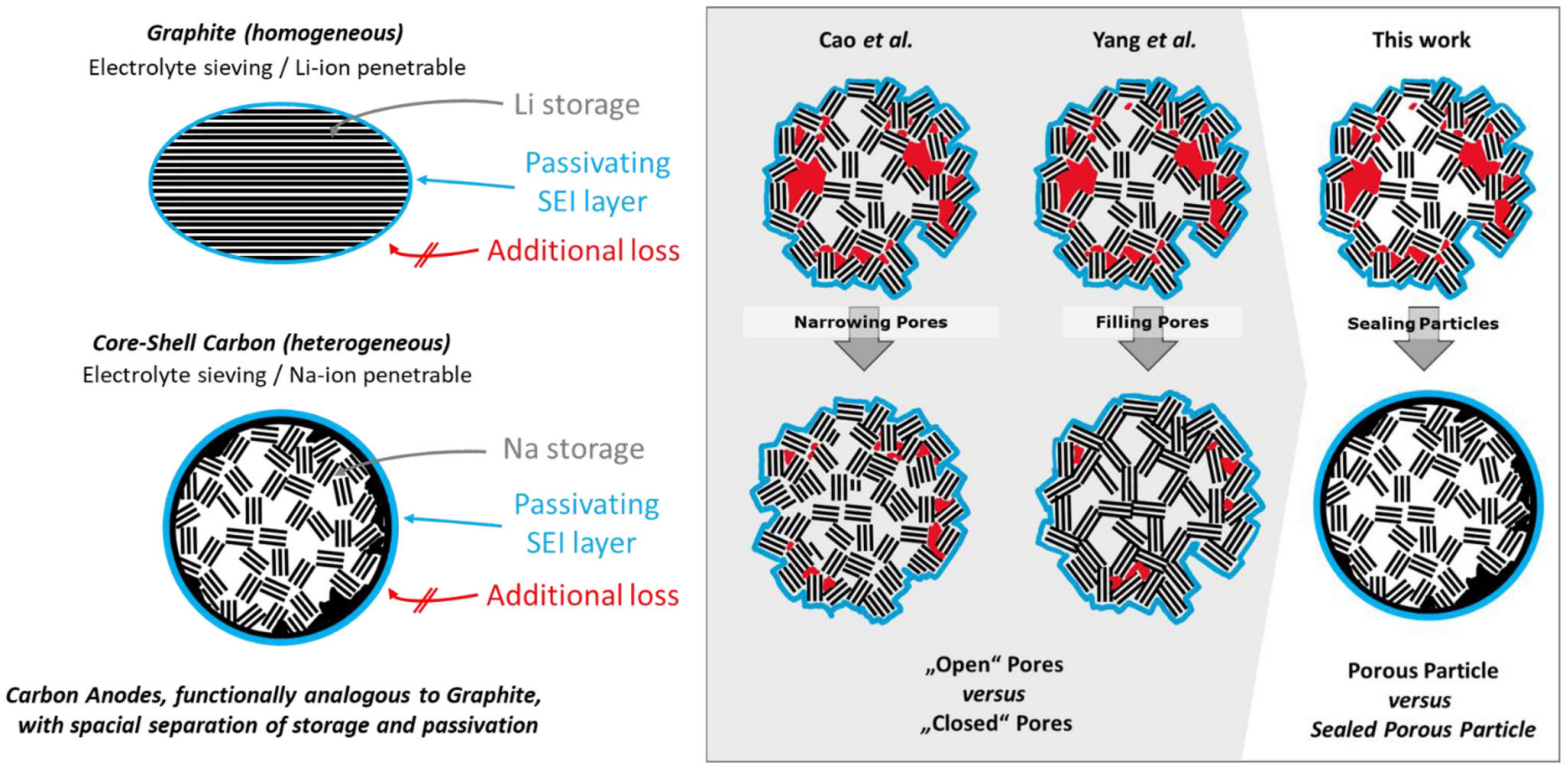
Paradigm shift of closed pores for reversible sodium storage in hard carbons. In graphite, both surface and bulk enable Li storage (Li^+^ penetrable, electrolyte sieving). In conventional hard carbons, the isotropic structure is penetrable to both Na^+^ and electrolyte, offering neither function. Core–shell carbons, with a porous core and sieving shell, combine these functions for Na storage.

Because of their low carbon footprint and high production rates, we utilize activated carbon as porous core material, similar to Cao et al. and Zhang et al.^[^ ^19^, ^20^ ^]^ Since our aim is to seal the particles with a semi‐permeable carbon coating while conserving porosity, we varied the type of activated carbon (see experimental part in SI) and adopted CVD conditions (unpublished experiments).^[^ ^20^ ^]^ Precursor choice and CVD method must be empirically found in order to successfully derive core‐shell materials. In this communication we focus on 1) characterization of Core vs. Core‐Shell material, 2) proof‐of‐concept that the heterogeneous particle structure improves SIB anode performance and 3) implications on current hypotheses of Na storage.

To confirm successful implementation, we characterize the structure and porosity of the core and compare it to the sealed materials. The porous core is denoted “Core,” the CVD‐coated derivative “Core‐Shell,” and the CVD product without a core, “Shell.” The process is schematically illustrated in Figure 1a. Sealing is carried out using toluene‐saturated N_2_ gas for three hours in a lab‐made CVD setup at 700 °C. Powder X‐ray diffractometry (PXRD) shows Core to be a highly disordered non‐graphitic carbon with low intra‐ and interplanar order, reflected by the weak (002) shoulder and broad (100) reflection at 2θ = 23°. The (002) feature, named according to graphite indexing convention, indicates interlayer distances centered around 3.9 Å, clearly above graphite (d = 3.34 Å), important for Na‐ion intercalation.^[^ ^24^ ^]^ Core‐Shell shows a 30% weight increase over Core and a higher degree of order with still broad but more defined (002) and (100) reflections at 2θ = 23° and 43°, respectively (Figure 1b). The Shell reference displays more intense peaks at 2θ = 23.7° and 43.7° (Figure 1c), indicating a more ordered structure, like the difference plot between Core and Core‐Shell (Figure 1c). Although Shell does not fully correspond to the material added in Core‐Shell, the changed structure seems largely due to addition of new carbon rather than modification of the Core. In addition, scanning electron microscopy (SEM) shows no evidence of a second phase: the particle morphology appears unchanged before and after CVD (Figure 1d,e), and no smaller “soot”‐like particles, typical of gas‐phase pyrolysis, are observed. The deviation of the Shell diffractogram from the difference plot may arise from different carbonization conditions or the difference between Core versus the tube surface as substrate, the latter providing a planar substrate that favors lattice alignment and experiencing higher local temperature.

**Figure 1:**
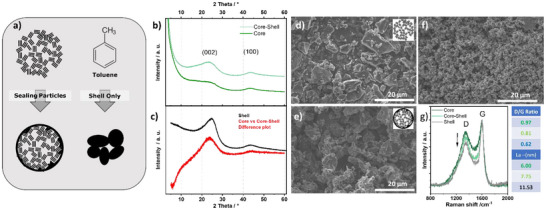
a) Schematic of the CVD process sealing particles with a thin carbon layer from pyrolyzed toluene and preparation of the “Shell” reference. b) XRD of Core and Core‐Shell. c) XRD difference plot (Core vs. Core‐Shell) compared with the Shell material d), e) and f) SEM images of Core, Core‐Shell and Shell. g) Raman spectra of Core, Core‐Shell, and Shell showing D and G bands with corresponding D/G ratios and calculated L_a_ values (fits in SI, Figure S2).

Raman spectroscopy was carried out to further elucidate the structural properties of the materials. The areal I_D_/I_G_ ratio was highest for the pristine Core (I_D_/I_G _= 0.97) and lowest for Shell (I_D_/I_G _= 0.62), meaning that Shell is more graphitic, in line with the XRD results. Core‐Shell shows a value of I_D_/I_G _= 0.81. The corresponding in‐plane graphitic domain sizes (L_a_) are ∼6.0 nm for the Core and ∼11.5 nm for the Shell. By calculation the deposition of Shell onto Core without affecting Core (1), extending L_a_ assuming sheets of the area A = L_a_^2^ domains (2) or adding new filling the pores with additional L_a _= 2 nm domains would result in 7.27, 6.84 and 5.4 nm if 30% weight increase is considered. The actually measured L_a_ for Core‐Shell is 7.75 nm, is closest to 1). This indicates that Core‐Shell may indeed be a composite material with a heterogeneous structure and the increased domain size is mainly explained as the average domain sizes of both components.

The CVD process clearly modifies the core particles, while Raman indicates sealing over filling of particles. Based on N_2_ adsorption–desorption porosimetry analyzed with the QSDFT model for carbon, Core shows a high SSA of 2690 m^2^ g^−^^1^, a total pore volume (TPV) of 1.18 cm^3^ g^−^^1^, and a pore size distribution with a maximum at d = 2.6 nm (Figure 2b). The isotherm points to a good transport pore system with no indications to bottleneck pores, since adsorption and desorption show no hysteresis over the entire p/p_0_‐range (Figure 2c). For Core‐Shell, the apparent SSA drops drastically to 14 m^2^ g^−^^1^, i.e., by a factor of 192 (Figure 2c). This value agrees well with the external surface area (ESA) of Core from the *t‐plot* method (Table S1), indicating that the measured area corresponds to the geometric particle surface after coating and thus confirms shell formation.^[^ ^25^ ^]^ The TPV corresponds to a porosity of 68% or 71% assuming skeletal densities of ρ = 1.8 g cm^−^^3^ or ρ = 2.1 g cm^−^^3^, respectively (calculation in SI). Assuming the same skeletal density for the shell, adding 30 wt% carbon inside the pores, would lower the porosity to 58% or 63%, with remaining TPVs of 0.78 or 0.80 cm^3^ g^−^^1^. By contrast, the measured TPV of Core‐Shell is nearly negligible (0.01 cm^3^ g^−^^1^), which would correspond to deposition of 211 wt.% or 246 wt.% assuming pore filling. The Shell reference material is a nonporous carbon, according to N_2_ sorption showing an SSA of 2.7 m^2^ g^−^^1^ (Figure 2d). The shell could not be directly visualized even by high‐resolution TEM (Figure S3). However, simple geometric considerations allow an estimate of shell thickness. Assuming spherical particles and a density of ρ = 1.8 g cm^−^^3^, the SSA of 14 m^2^ g^−^^1^ corresponds to an average particle radius of ∼314 nm (calculation in SI); for ρ = 2.1 g cm^−^^3^, the radius is ∼297 nm. From this model, the mass increase equates to a shell thickness of ∼9 nm or ∼8 nm.

**Figure 2:**
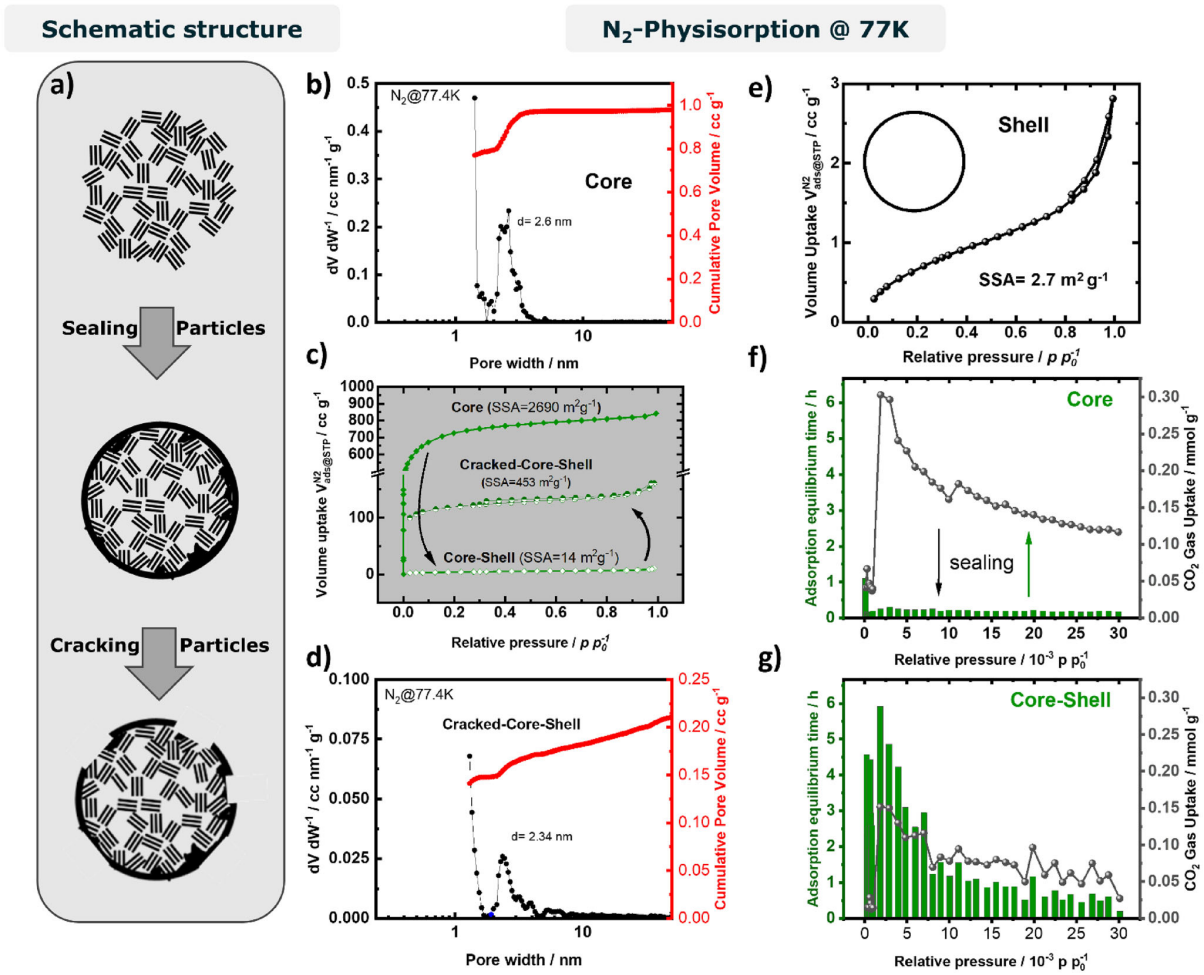
a) Schematic of the CVD sealing process and subsequent “cracking” of Core‐Shell particles by ball milling. b, d) N_2_ sorption pore size distributions of Core and “Cracked‐Core‐Shell”. c) Comparative N_2_ adsorption–desorption isotherms before/after CVD coating and after cracking. e) N_2_ sorption of the Shell material. f, g) CO_2_ adsorption measurements showing equilibrium times and corresponding gas uptake at individual points.

Such a dimension is plausible and likely permeable due to inhomogeneities, while it also explains why the shell is difficult to observe on a rough material. Furthermore, even “nonporous” carbons contain ultramicropores (<0.7 nm). A thin ultramicroporous film may therefore allow limited diffusion of sorption gases despite shell coverage. It was previously observed that SSA values for hard carbons vary strongly with the probe molecule used for sorption.^[^ ^4^, ^26^, ^27^ ^]^ For the same material, N_2_ and CO_2_ sorption can deviate by more than a factor of 200.^[^ ^26^ ^]^ We therefore additionally characterized Core and Core‐Shell by CO_2_ porosimetry (Figure 2f,g), applying long equilibration times at each pressure point. Core shows a higher CO_2_ uptake (TPV = 0.375 cm^3^ g^−^^1^), while Core‐Shell reaches ∼50% of this value (TPV = 0.188 cm^3^ g^−^^1^), confirming significant residual porosity, which is not accessible by N_2_ at T = 77.4 K. The CO_2_‐based SSAs are 989 m^2^ g^−^^1^ for Core and 475 m^2^ g^−^^1^ for Core‐Shell. For Core, the gas uptake increases steadily with relative pressure, whereas Core‐Shell shows strong fluctuations (Figure 2g), indicating kinetic hindrance and high diffusion resistance. This is supported by equilibration times: ∼15 min per point for Core versus > 1 h, often up to 4 h, for Core‐Shell. These results point to successful preparation of core–shell carbons with a porous core and a semi‐permeable shell, penetrable only through ultramicropores by small gas molecules at 25 °C.

To further test whether a coating is present, we attempt to reverse the sealing by ball milling. After milling (the milled sample is referred to as “Cracked‐Core‐Shell”), N_2_ sorption porosimetry was repeated and compared to pristine Core (Figure 2b–d). The N_2_‐based SSA increased from 14 to 453 m^2^ g^−^^1^, indicating that indeed a shell is covering a high‐surface area material in the core of Core‐Shell (Figure 2c). In the cases of homogeneously narrowed pore entrances or homogeneous filling of the pores, one would not expect significant changes in surface area in case of destruction of particles, due to a remaining inaccessibility of the pores to probe gases. Pore size distributions of Core and Cracked‐Core‐Shell are very similar, with a minor decrease in average pore size from 2.60 nm to 2.34 nm (Figure 2b and d), which may indicate core domain growth or filling to a minor extent. Its SSA is lower than pristine Core, likely due to incomplete cracking and/or partial modification of the porous core during CVD (see slightly smaller pores in Cracked‐Core‐Shell). Consistently, reutilized pore volume (0.38 cm^3^ g^−^^1^ vs. 1.18 cm^3^ g^−^^1^ for Core) is much smaller than the original, but still more than an order of magnitude higher than expected for filled particles. In contrast to the original isotherm, a slight hysteresis is observed. This may indicate the formation of bottlenecks between pores, but may also be due to remaining shell, hindering desorption. Interestingly, the recovered relative pore volume (∼32%) exceeds the SSA recovery (∼17%). Cumulative pore volume plots (Figure 2b, d) reveal additional mesopore contributions after milling, which explains the additional pore volume. These likely arise from meso‐sized particles formed during milling, which agglomerate to create interstitial voids detected as mesopores. SEM confirms this, showing many meso‐sized particles not observed before milling (Figure S4).

With evidence for core–shell carbon anodes consisting of a porous core and a semi‐permeable shell, electrochemical sodiation/desodiation tests are performed. The Na‐storage behavior of Core, Shell, and Core‐Shell is analyzed by triplicate measurements in lab‐made coin cells (details in experimental section). As expected, the highly porous Core shows poor performance (Figure 3a), with a first‐cycle loss of 636 ± 28 mAh g^−^^1^ (ICE 17 ± 1%) and a reversible capacity of 134 ± 1 mAh g^−^^1^ over the next four cycles (Figure 3a′). The Shell reference also shows limited performance (Figure 3b), despite its low SSA of 2.7 m^2^ g^−^^1^, showing a first‐cycle loss of 246 ± 31 mAh g^−^^1^ (ICE 34 ± 3%) and 127 ± 7 mAh g^−^^1^ reversible capacity over four cycles (Figure 3b′). Both materials exhibit only sloping profiles without a low‐voltage plateau (LVP). The low ICE‐value of Shell, is a consequence of low reversible storage capacity, rather than the only moderately increased irreversible loss (see SI equation 1). After CVD sealing, the material shows outstanding electrochemical behavior. The first‐cycle loss decreases by a factor of 7 to 89 ± 30 mAh g^−^^1^, while the reversible capacity almost triples to a very high capacity of 400 ± 24 mAh g^−^^1^. The capacity of 400 ± 24 mAh g^−^^1^ therefore comes with a commercially relevant ICE of 82 ± 2%, both clearly outperforming the current commercial state‐of‐the‐art (see Figure S5). For the following four cycles, Coulomb efficiency (CE) exceeds 99%, which promises possible high cycle lifetimes, if the cell design is further optimized. These results confirm that sealing porous particles greatly enhances ICE, demonstrating the promise of our core–shell anode strategy based on a thin non‐graphitic carbon shell. In graphite LIB anodes, low ICE in the first cycle stems from SEI formation on the external particle surface.^[^ ^17^ ^]^ Similarly, sealing Core creates an external particle surface for SEI formation, spatially separating it from the porous core. Unlike traditional hard carbons, the core‐shell strategy eliminates the complexity of optimizing the pore shape and composition toward solvent interactions.

**Figure 3:**
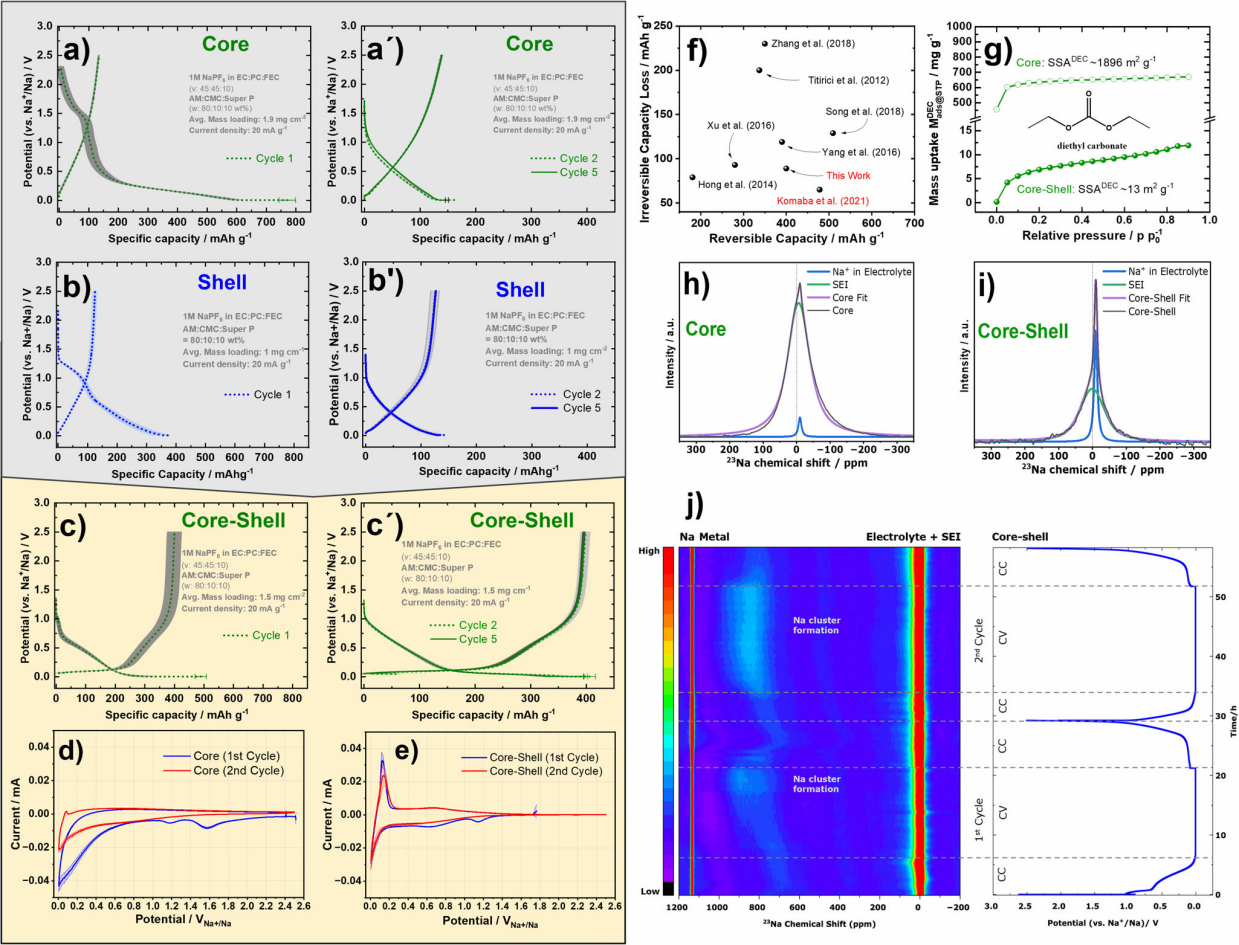
a–c′) Electrochemical performance of the core, shell, and core–shell carbons including first cycle galvanostatic discharge/charge curves of core a), shell b), core–shell c) and corresponding voltage profiles for subsequent cycles (2 and 5) highlighting capacity retention (a′–c′). d),e) Cyclic voltammograms of Core d) and Core‐Shell e). f) Literature comparison of Hard carbon materials. g) DEC DVS adsorption–desorption isotherms at 298 K showing the high specific surface area of the core (1896 m^2^ g^−^^1^) and the reduced surface area after CVD coating in the shell (13 m^2^ g^−^^1^). h),i) Ex‐situ ^23^Na NMR spectroscopy of cycled Core and Core‐Shell showing the characteristic SEI peak at ‐10 ppm. j) Operando ^23^Na solid‐state NMR spectra of an electrochemical half‐cell comprising core–shell carbon, sodium metal, and NaPF_6_ electrolyte. The corresponding electrochemistry for the 1^st^ and 2^nd^ cycles is shown on the right‐hand side of the spectrum.^[^ ^19^, ^21^, ^28^, ^32^, ^33^, ^34^, ^35^ ^].^

To gain general mechanistic understanding of reversible and irreversible processes, first and second cycle sodiation behavior was investigated by half‐cell galvanostatic cycling (Figure 3a–c′) in combination with cyclic voltammetry (CV) (Figure 3d and e). The electrochemical characteristics differ strongly between Core, Shell, and Core‐Shell. For Core, three contributions are observed: 100 mAh g^−^^1^ between 2.25–1.25 V (irreversible peak at 1.58 V), 100 mAh g^−^^1^ at 1.25–0.355 V (irreversible peak at 1.18 V), and the largest part below 0.355 V (broad irreversible shoulder), mainly from reductive PC decomposition and extensive SEI formation.^[^ ^28^, ^29^ ^]^ Apparently, the SEI formation in Core consumes most of the capacity, since the remaining reversible capacity extracted through desodiation is far smaller (134 ± 1 mAh g^−1^) than in Core‐Shell (400 ± 24 mAh g^−1^). The reductive electrolyte decomposition in Core fills potential storage pores, therefore the following sodiation/desodiation curves (Figure 3a′) severely underestimate the core‐material‘s inherent ability to store sodium and interfere with the adsorption of Na into pores, eliminating the possibility to run into a plateau. Shell shows only two contributions: No significant feature above 1.25 V, ∼130 mAh g^−^^1^ at 1.25–0.60 V and ∼240 mAh g^−^^1^ below 0.6 V. The absence of a high‐potential region may be due to its more graphitic character, consistent with fewer functional groups. Core‐Shell at higher potentials interestingly resembles Shell, while it additionally comprises an extended LVP storage capacity. There is no significant feature above 1.25 V, suggesting that high‐potential reactions are not solely due to Na^+^–functional group interactions within the hard carbon anode particles, but point to required electrolyte solvent molecules as reaction partner. A feature with ∼6 mAh g^−1^ between 1.25–1.00 V (irreversible peak 1.16 V) and one with ∼90 mAh g^−^^1^ between 0.65 and 0.44 V (irreversible peak at 0.65) are observed. At lower potential (below 0.2 V) a kinetic current is observed in the CV, which relates to the LVP. Since no graphitization of the core material was involved, this finding is interesting and sparks curiosity if pseudo‐metallic Na clusters are therein formed.^[^ ^13^ ^]^ Comparison of the reversible cycling profiles of Core, Shell, and Core‐Shell is even more revealing. The Core/SEI composite, resulting from the highly irreversible first cycle, shows the typically observed sloping profile, with sodiation up to 1.75 V and desodiation up to 2.5 V. The Shell/SEI composite has a similar reversible capacity but a distinctly different shape: sodiation starts at 1.27 V, and the desodiation slope is negligible above 1.1 V, indicating negligible high‐potential desodiation. Curiously, Core‐Shell, like in the first cycle, resembles Shell, but with the additional LVP. This comparison suggests that sloping capacity arises from carbon/SEI composites (becoming more pronounced with higher concentrations of functional groups), whereas the LVP stems from sodium adsorption in carbon without electrolyte interference. Contrary to current hypotheses, these results indicate that plateau capacity does not primarily depend on graphitization degree; instead, it is compromised when electrolyte decomposition products occupy the pore system like observed in Core.

While this study aims at the proof‐of‐concept, an initial assessment of cycle life stability and rate performance was carried out in half‐cell tests with the same loading of ∼1.1 mg cm^−2^ (Figure S6). While the loading allows consistent comparison, the resulting relatively low absolute currents will also result in relatively harmless resistances. After 5 cycles at C/20 an increase in C‐rate to C/2 results in a successive capacity drop from 406 ± 20 to 246 ± 16 mAh g^−1^, and a stabilization at ∼177 mAh g^−1^. After 50 cycles at C/2 98% of the capacity at C/20 can be recovered indicating integrity of the Core‐Shell particles. Rate tests show that this is also the case after cycling as fast as 5C. At C/10 ∼85% of the original capacity are retained, at 2C the electrode still delivers 25%. Overall, the tests indicate good rate performance and cycling stability, although cycle stability could still be improved with optimized cell design.

Spatial separation of storage and SEI formation seem to be a successful concept to break the undesirable trend of large reversible capacities being tied to high irreversible capacity (Figure S1 and 3f). If the SEI formation on such core‐shell particles is forced to the external surface (shell), the irreversible losses should be proportional to the SSA of the material, as it is the case for graphite anodes in LIBs.^[^ ^17^ ^]^ While the ICE of the herein presented Core‐Shell with 82 ± 2% is already promising, further reduction of the ESA would be a way to minimize losses. But also for graphite in LIBs, high ICEs are only reached for very small ESAs of ∼2 m^2^ g^−1^.^[^ ^4^ ^]^ However, since surface area measurements with small probe molecules like N_2_ and CO_2_ are not reliable for hard carbons, we herein test dynamic vapor sorption (DVS) with diethyl carbonate (DEC), a representative carbonate electrolyte solvent molecule, as alternative technique to quantify the liquid electrolyte interface (LEI). At 298 K Core shows a DEC uptake of 670 mg g^−^^1^ at 90% relative pressure, which is drastically reduced to 12 mg g^−^^1^ for Core–Shell (Figure 3g). Calculating SSA from these isotherms requires the molecular cross‐sectional area. Therefore, we carried out grand canonical Monte Carlo simulations of DEC adsorption on monolayer graphene, yielding an effective footprint of 0.61 nm^2^ per molecule (Figure S8). Using this footprint, the DEC uptake at low saturation pressure (5%) corresponds to a SSA of 1896 m^2^ g^−^^1^ for Core, reduced to only 13 m^2^ g^−^^1^ for Core–Shell (Figure 3g; detailed calculations in SI). Apparently, DEC is too large to penetrate the shell and electrolyte solvent vapor sorption is a promising method to quantify the LEI of hard carbons. The results suggest that the significant improvement of the reversible capacity of the core‐shell material is linked to particle sealing with a semi‐permeable carbon layer. By the quantification of the LEI, the method allows to directly relate irreversible losses for hard carbons to the external surface area, i.e., particle geometry. This implies that like for graphite in LIBs, the irreversible loss of hard carbon anodes is due to SEI formation.

In order to validate this implication, we carried out ex‐situ solid‐state ^23^Na NMR spectroscopy measurements on fully sodiated Core and Core‐Shell (Figure 3h and 3i). Ionic sodium species, related to residual electrolyte salt or SEI, are diamagnetic and observed at low chemical shifts. The mobile free electrolyte appears with a sharp signal at a chemical shift of ‐10 ppm and while the precipitated SEI or carbon surface‐adsorbed Na species produce a broad signal at + 1 ppm.^[^ ^30^, ^31^ ^]^ While Core shows a large and broad signal, composed of an SEI‐related peak, with a much higher intensity compared to the free electrolyte, the signal for Core‐Shell remains sharp, pointing to much less significant formation of such species. The suppression of this broad signal in Core‐Shell shows that the shell restricts SEI formation within the pores. We can conclude that the shell indeed reduces the irreversible loss due to a suppressed SEI formation, caused by its electrolyte solvent impermeable nature. The SEI formation can therefore be directly related to the LEI measured via electrolyte solvent sorption. The often excessive irreversible loss for hard carbons is explained by electrolyte decomposition within hard carbon particles caused by electrolyte permeation into the particles. This SEI filling alters the properties of the HC into a composite material, covers the Na storage capacity of the HC and results in the capacitive sodiation/desodiation profile with reduced Na storage capacity.

To investigate the Na‐storage mechanism of the Core–Shell and Core materials, operando solid‐state ^23^Na NMR spectroscopy was performed (Figure 3j and Figure S7, respectively), taking advantage of its ability to probe the different Na‐containing local atomic environments present during electrochemical cycling. Two distinct peaks are consistently observed in both systems during cycling: the resonance centered around − 10 ppm assigned to diamagnetic sodium ions, including contributions from the NaPF_6_ electrolyte as well as Na^+^ in the bulk electrode and SEI layer, and a resonance at 1135 ppm attributed to metallic Na from the sodium‐metal counter electrode.

Notably, during sodiation the Core–Shell material exhibits an additional resonance at around 852 ppm, which coincides with the plateau region in the galvanostatic profile. This resonance arises from a Knight shift and is attributed to the formation of quasi‐metallic sodium clusters, consistent with cluster formation within the pore structure as reported in operando and ex situ NMR studies of hard carbons.^[^ ^30^, ^31^ ^]^ In contrast, no such feature is observed for the core material (Figure S7). The cluster formation in the core‐shell material is reversible, appearing in both the first and second cycles. While the cluster formation for the nongraphitized Core‐Shell is interesting by itself, it is further curious that the quasi‐metallic Na in the second cycle is clearly related to the plateau of the constant voltage step at 5 mV, while this is not the case for the first cycle. Apparently, the first cycle is crucial for the formation of passivating SEI, but there seems to be an additional “formation” of the carbon core, necessary to be able to form pseudo‐metallic clusters.

In summary, we demonstrated that a CVD process can be utilized to deposit a non‐graphitic carbon shell onto highly porous activated carbon, while largely conserving core porosity, thereby converting it into a well‐performing Na‐ion anode material. The porous core of the materials remains partially accessible to CO_2_ at 298.15 K but not to N_2_ at 77 K, confirming the semi‐permeable function of the shell. Porosity analysis after mechanically deconstructing the core‐shell material revealed a nearly unchanged pore size of the original pores. The sealing of the external surface of carbon anode particles results in significantly reduced irreversible losses (ICE 82 ± 2%) and increased reversible capacities 400 ± 24 mAh g^−^^1^. The combination of diethyl carbonate vapor sorption and ex situ ss‐NMR spectroscopy indicates that the sealing is impermeable to electrolyte solvent molecules supressing undesirable SEI‐formation within the particles. Evaluation of the core material shows that this SEI‐filling of hard carbon particles causes enhanced irreversible losses. The semi‐permeable shell creates a distinct liquid electrolyte interface (LEI), which may prospectively be quantitively related to SEI formation like the BET surface area of graphite to the SEI‐loss in lithium‐ion batteries.

These findings support our core–shell strategy: while both storage and molecular sieving are essential, hard carbons cannot efficiently combine them in one homogeneous phase. A semi‐permeable shell forces electrolyte reduction and SEI formation to the external surface, while the guarded core provides effective storage. The emergence of a plateau without high‐temperature treatment suggests that defective carbons can host pseudo‐metallic sodium clusters, which was also supported by operando‐electrochemical ss‐NMR spectroscopy. The observation that the pseudo‐metallic signal is developed throughout the CV‐step at 5 mV in the first cycle, indicates that not only the liquid‐electrolyte interface needs to be passivated (SEI formation), but also the carbon‐sodium interface requires passivation for the formation of pseudo‐metallic Na. Interestingly, neither a highly graphitized carbon nor specifically shaped pores are required to address the adsorption‐type Na storage mechanism.

## Supporting Information

The authors have cited additional references within the Supporting Information.^[^ ^15^, ^19^, ^21^, ^32^, ^33^, ^34^, ^35^, ^36^, ^37^, ^38^, ^39^, ^40^, ^41^, ^42^, ^43^, ^44^, ^45^, ^46^, ^47^ ^]^

## Conflict of Interests

The authors declare no conflict of interest.

## Supporting information



Supporting Information

## Data Availability

The data that support the findings of this study are available from the corresponding author upon reasonable request.
