# Supplementary material for: Core‐Shell: Resolving the Dilemma of Hard Carbon Anodes by Sealing Nanoporous Particles With Semi‐Permeable Coatings
Source: Angew Chem Int Ed Engl. 2026 Jan 22;65(10):e19457. doi: 10.1002/anie.202519457 (PMC12955508; doi:10.1002/anie.202519457)
Supplement: Supplementary file 1 — Supporting Information [file ANIE-65-e19457-s001.pdf]

Supporting Information

Material Synthesis:

The nanoporous carbon used as core material was the activated carbon “Carbopal SC 11 CAT” from Donau Carbon (Germany). Core-Shell material was prepared by heating the core material to a temperature of 700°C in a horizontal tube furnace with a heating rate of 5°C min^{1} under an argon atmosphere (1 L min^{-1}). At the target temperature the gas flow was switched to toluene-saturated N_2 at the same flow rate for 3 hours. The core sample weight was 1.0 g. Due to lack of carbon formation on the tube surface at 700 °C, Shell was extracted after CVD at higher temperatures ie. 900°C

Ball Milling:

Core-shell material was ball milled using a Retzsch MM 400 ball swing mill. 100 mg of the material was placed in 10 ml ZrO_2 jars with two 1 mm ZrO_2 balls. The material was ball milled for 3x3 min at 25 Hz.

Material Characterisation:

N_2 gas sorption analysis was performed using Quantachrome Autosorb iQ-C-MP and NOVA instrument (Anton Paar, Austria). All samples were outgassed under turbopump vacuum at 200°C for 12 h before measurements. Nitrogen sorption measurements were completed at 77.4 K. The pore volumes and specific surface areas (SSA) were obtained from N_2 sorption data with a QSDFT slit cylindrical adsorption model.

CO_2 gas sorption analysis was performed using a Micromeritics TriStar (Micrometrics, USA). All samples were outgassed under turbopump vacuum at 200°C for 12 h before measurement. CO_2 sorption measurements were completed at 298.15 K. The pore volume and specific surface area (SSA) were obtained using a CO_2 NLDFT-slit model from the adsorption branch.

Powder X-ray diffraction (PXRD) was collected using a D8 Advance diffractometer (Bruker AXS, Germany) equipped with an energy-dispersive LynxEye XE-T detector. Powder samples were prepared on flat sample holders for analysis. The measurements utilized a copper X-ray source (wavelength: 1.54178 Å) without a monochromator. Experiments were carried out in Bragg-Brentano geometry, with the X-ray tube operating at 40 kV and 40 mA. The measurement parameters included a 2θ range of 3–60°, a step size of 0.02°, a dwell time of 0.6 s per step, and a total acquisition time of 30 minutes.

Scanning electron microscopy (SEM) was performed using a Hitachi FlexSEM equipped with a tungsten (W) cathode and UVD detector. **TEM images** were taken by a JEM-ARM200CF (Jeol, Japan) probe Cs-corrected scanning transmission electron microscope (STEM) equipped with a cold field emission electron source operated at 80 kV.

Raman spectra were collected using a Renishaw inVia confocal Raman microscope (Renishaw, UK) equipped with a 532 nm laser in backscattering geometry. The laser power was attenuated to 1% of the nominal output (1 mW at the sample surface) to minimize local heating effects. Measurements were performed in the range of 200–2500 cm^{-1} using a grating with a spectral resolution of ~1 cm^{-1}. Each spectrum was acquired with an integration time of 1 min and averaged over three accumulations. Two spectra were recorded at randomly selected positions on each powder sample to ensure reproducibility. Spectral deconvolution was carried out by fitting the Raman bands with Lorentzian functions. The fitting was performed after baseline subtraction, and parameters such as peak position, intensity were extracted. The $\frac{I_D}{I_G}$ ratio was calculated using the integrated area of the D and G Peaks The length of the graphitic domain was calculated using $L_A = (2.4 \times 10^{-10}) * \lambda^4 * \left(\frac{I_D}{I_G}\right)^{-1}$.

Dynamic vapour sorption experiments were carried out using a DVS Resolution Vapor Sorption Device (Surface Measurement Systems, UK) with diethyl carbonate (DEC) vapor as the sorbate (Sigma Aldrich, Germany). The desired relative vapor pressure was achieved by controlled mixing of dry and DEC-saturated carrier gas streams within the instrument. To complement the experiments, Grand Canonical Monte Carlo

simulations were carried out using the RASPA simulation package to model DEC adsorption on monolayer graphene, using 20000 initialization steps and 100000 sampling steps.[31] A rectangular graphene unit cell with unit cell parameters a = 3.4548 nm, b = 3.4194 nm was used, corresponding to a 14x8 supercell of the primitive rectangular unit cell. The interlayer distance was c = 10 nm, much larger than the Van-der-Waals cutoff distance of 1.2 nm. The Lennard-Jones potential of graphitic carbon was set to σ = 0.34 nm and ε/k_b = 28 K.[32-35] For diethyl carbonate, we used the structure and the Transferable Potentials for Phase Equilibria Force (TraPPE) parameters from Luo *et al*.[36] Inter-atomic Lennard-Jones potentials were derived using the Lorentz-Berthelot mixing rule.[37] The simulation temperature was set to 298.15 K and pressures in intervals of 66.5 Pa from 66.5 Pa – 1263.5 Pa were sampled, corresponding to approximately 5 – 95% of the saturation vapor pressure of DEC at room temperature ($p_0 \approx 1330$ Pa).[38]

Electrochemical characterization was undertaken using CR2032 type coin cells, which were assembled in an Ar-atmosphere glovebox with the stack consisting of metallic sodium chips (AOT, China), two glass fiber separators Type 691 (VWR, Germany) and the carbon electrode. The electrolyte used was 100 µl of 1M $NaPF_6$ in EC:PC:FEC (45:45:10, v.%, E-Lyte, Germany). Electrodes were punched from a 80:10:10 active material, CMC, Carbon Black coating, coated on carbon coated aluminium foil (AOT, China). Electrochemical testing was performed on Biologic VMP3e and MPG2 potentiostats (Biologic, France). Galvanostatic sodiation/desodiation was performed between 2.5 V and 5 mV at a current density of 20 mA g^{-1}, consisting of constant current (CC)/ constant voltage (CV) steps during sodiation with a voltage hold at 5 mV until the current dropped below 10 uA mg^{-1} and CC-only steps during sodiation. The ICE is calculated according to:

$$\mathrm{ICE}=\frac{C(sodiation)}{C(desodiation)}\cdot 100=\left(1-\frac{C(irreversible)}{C(desodiation)}\right)\cdot 100 \quad \text{(equation 1)}$$

Cyclic voltammetry was performed by sweeping the potential linearly from 2.5 V to 5 mV at a scan rate of 0.1 mVs^{-1} with the current recorded every 0.1 s. Long-term cycling stability was evaluated galvanostatically within the same voltage window. The cells were cycled at C/20, for five cycles, followed by 50 cycles at C/2 and finally three cycles at C/20 to assess reversibility. The cutoff current was defined as half the applied c-rate. Rate capability was investigated by cycling at various c-rates (C/20, C/10, C/5, C/2, 1C, 2C, 5C) under the same cutoff criterion, with five cycles performed at each rate.

Ex Situ Hahn-echo NMR spectra were acquired at room temperature on a Bruker Avance 400 NMR spectrometer (^{23}Na 105.86 MHz). 2.5 mm zirconia rotors were measured under static conditions. NaF was used as a reference for ^{23}Na (7.4 ppm). The materials were cycled in half cell coin cells as powders against sodium metal, They were cycled to 5 mV and held at 5 mV. 1M $NaPF_6$ in EC/PC/FEC (45:45:10 vol%) was used as electrolyte. Samples were packed under argon atmosphere. The recycle delay was set to 0.1 s, 20000 transients were collected and the π/2 pulse length was set to 1.2 us.

Operando NMR measurements were performed on a Bruker Avance 500 MHz spectrometer (^{23}Na Larmor frequency = 132.3 MHz) fitted with a static probe system featuring automatic tuning and matching capabilities and connections for an external battery cycler. An in situ electrochemical cell from ePROBE was used in a cylindrical plastic cell capsule (outer diameter 11 mm) and aligned such that the electrodes were parallel to the applied magnetic field.

The in situ cells were assembled using a powder electrode as the working electrode and Na metal as the counter electrode, with 1M $NaPF_6$ in EC/PC/FEC (45:45:10 vol%) as the electrolyte. Cells were galvanostatically cycled over a voltage range of 2.5 V to 0.005 V vs. Na/Na^+ at a current density of 20 mA g^{-1}. At the end of each sodiation step (0.005 V), a constant-voltage hold was applied at the lower cut-off, limited to 20 h or until the current dropped below 2 mA g^{-1}. To maximise the signal-to-noise ratio within the restricted acquisition time, a one-pulse sequence was used. A 90° pulse corresponded to 5 µs at 200 W using an 11 mm diameter silver coil. For each spectrum, 30,720 transients were collected with a recycle delay of 0.05 s (32 min per spectrum). The ^{23}Na chemical shifts were referenced to a 1 M NaCl solution at 0 ppm.

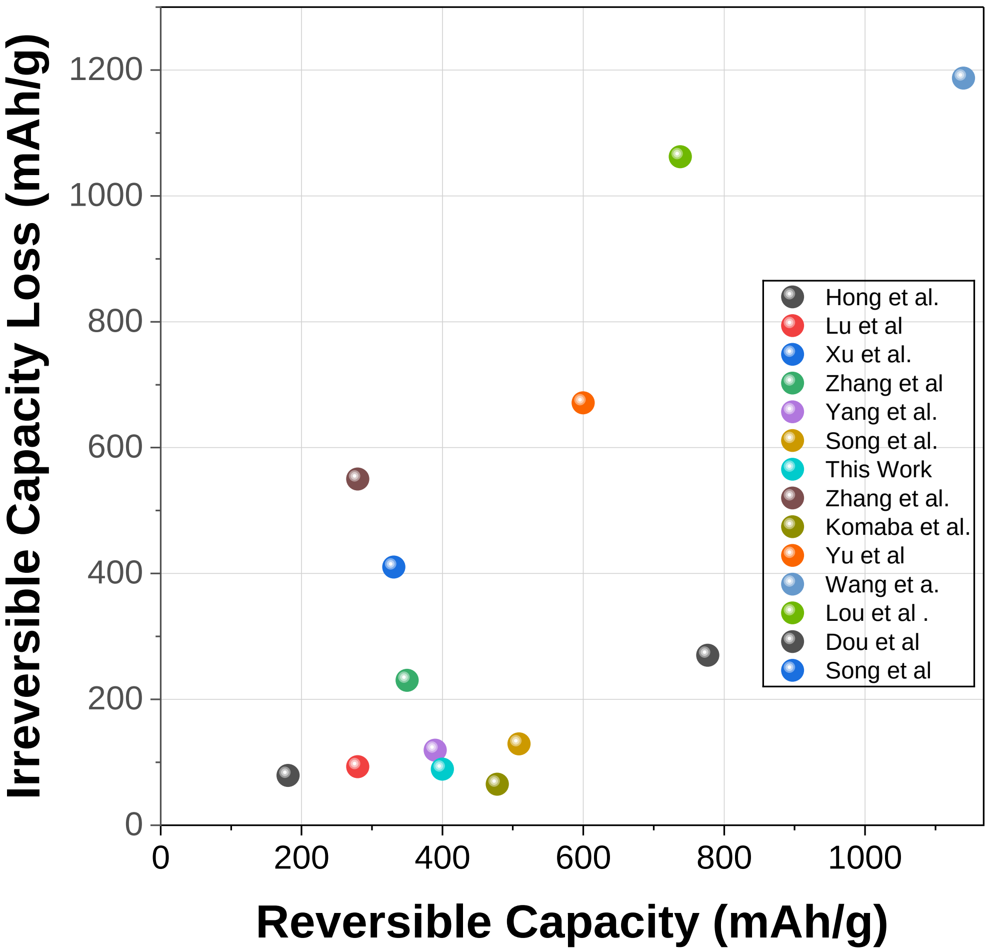

Figure S1. Literature comparison of irreversible capacity loss *versus* reversible capacity for various carbon-based anode materials in sodium-ion batteries, adapted from literature reports. [14-15,31-22,39-48]

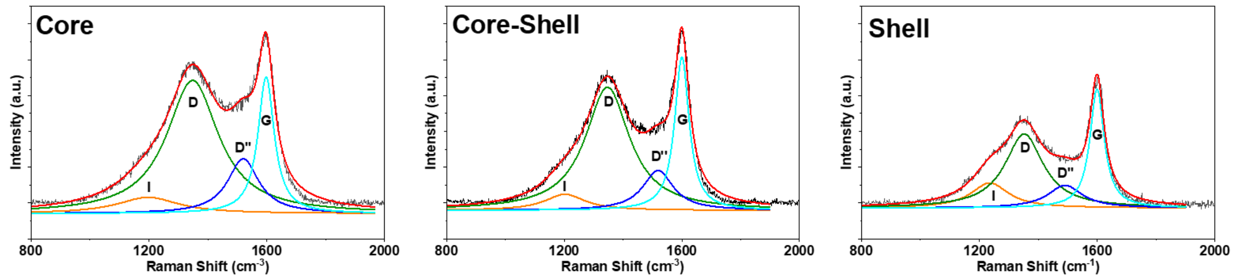

Figure S2. Raman spectra of Core, Core-Shell, and Shell materials in the range of 800–2000 cm^{-1}. The experimental data (black lines) are shown together with the overall fits (red lines), obtained using Lorentzian functions, and the individual deconvoluted peaks (coloured lines) corresponding to the D, G, I, and D″ bands.

Table S1. External surface area of Core determined by N_2 gas sorption analysis calculated by t-plot method.

Material	External SSA (m^2/g)
Core	14

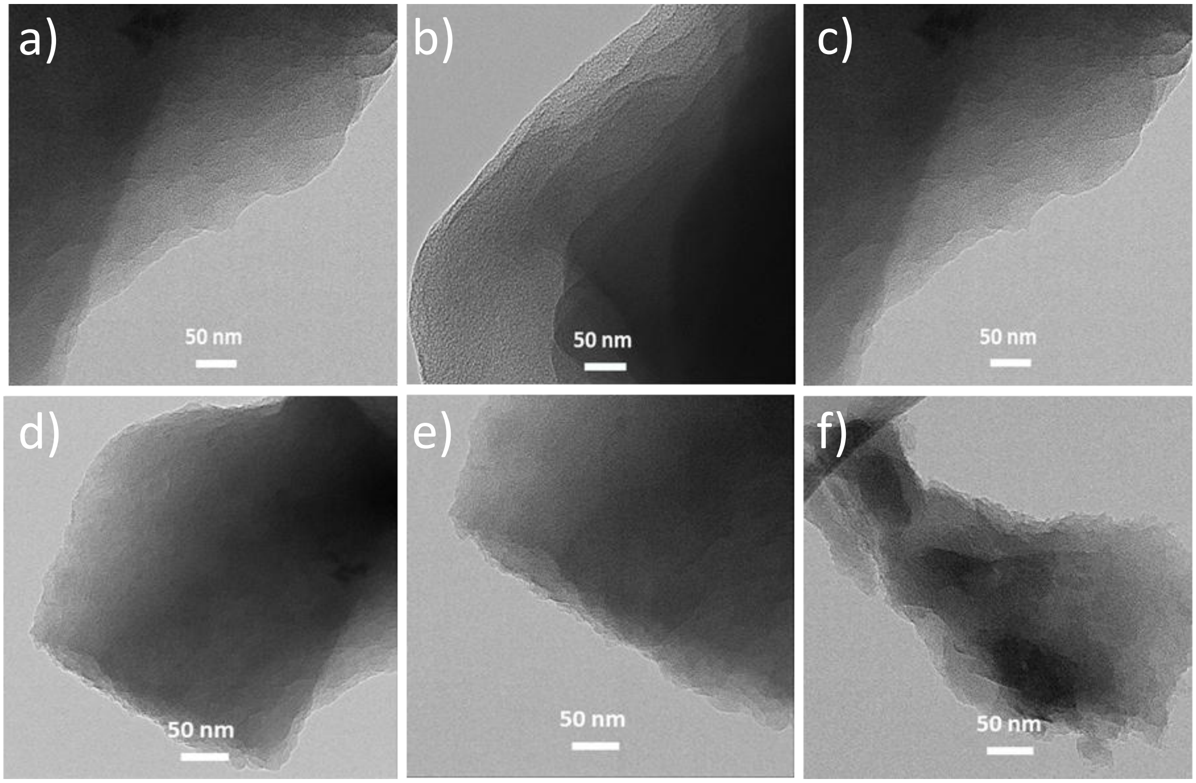

Figure S3. TEM images of Core (a-c) and Core–Shell (d-f) materials.

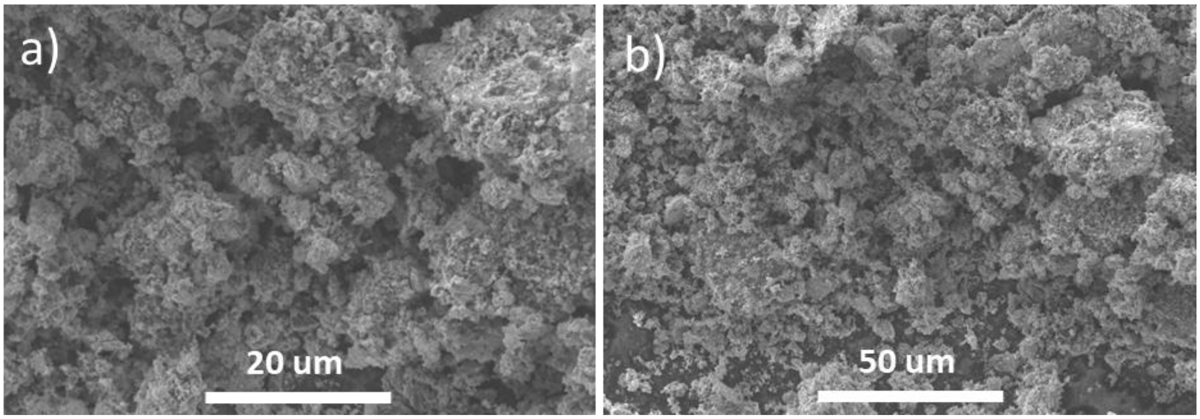

Figure S4. SEM images of ball-milled core–shell materials showing mesoscopic particles arising from the mechanical break-down.

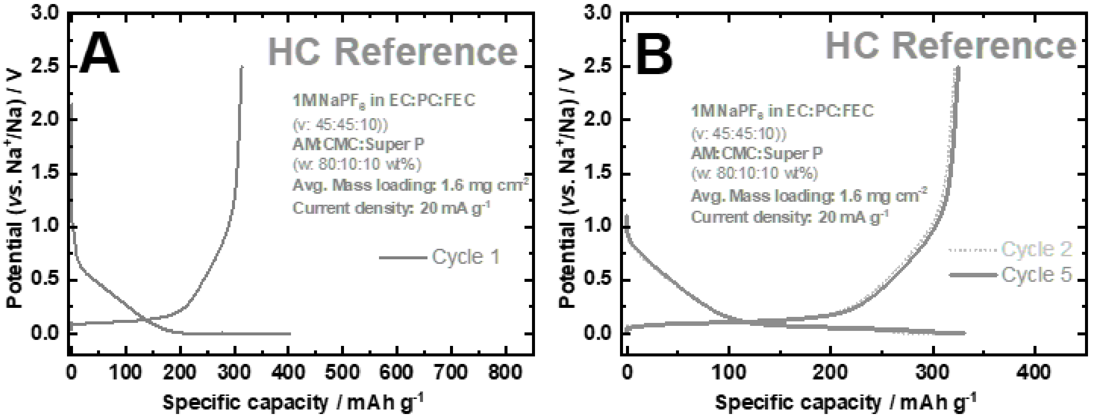

Figure S5. First cycle galvanostatic sodiation/desodiation curves of HC reference electrodes in half-cell measurements (a) and corresponding voltage profiles for subsequent cycles 2 and 5 (b).

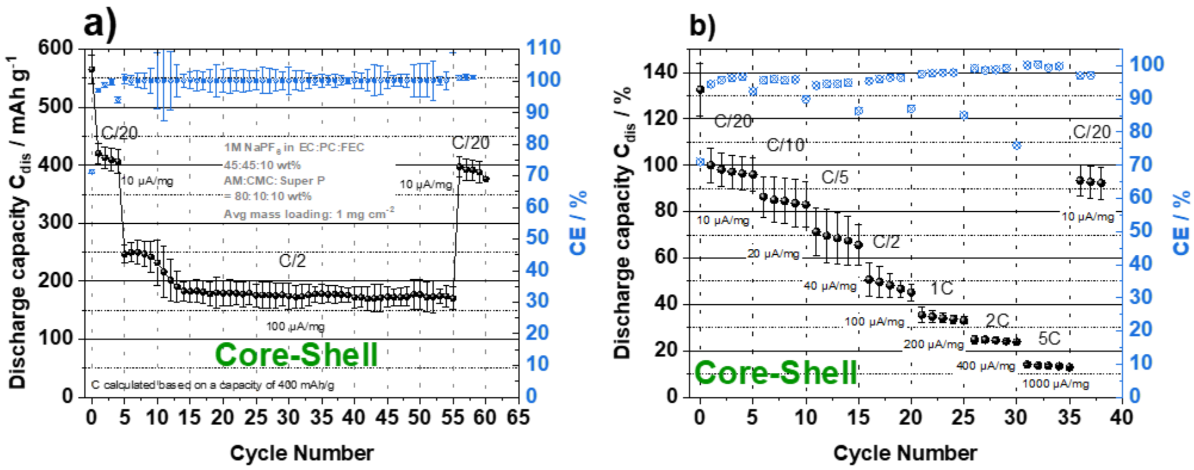

Figure S6. Cycling stability (a) and rate tests (b) of the Core–Shell electrode in half-cell measurements, showing discharge capacity and Coulombic efficiency vs. cycle number. Electrode composition and electrolyte for b) mirrored that of a) with avg. mass loading of 0.7 mg cm^{-2}. C-rate for CC-step and current cutoff for CV-step are shown. The cutoff current was defined as half the applied C-rate.

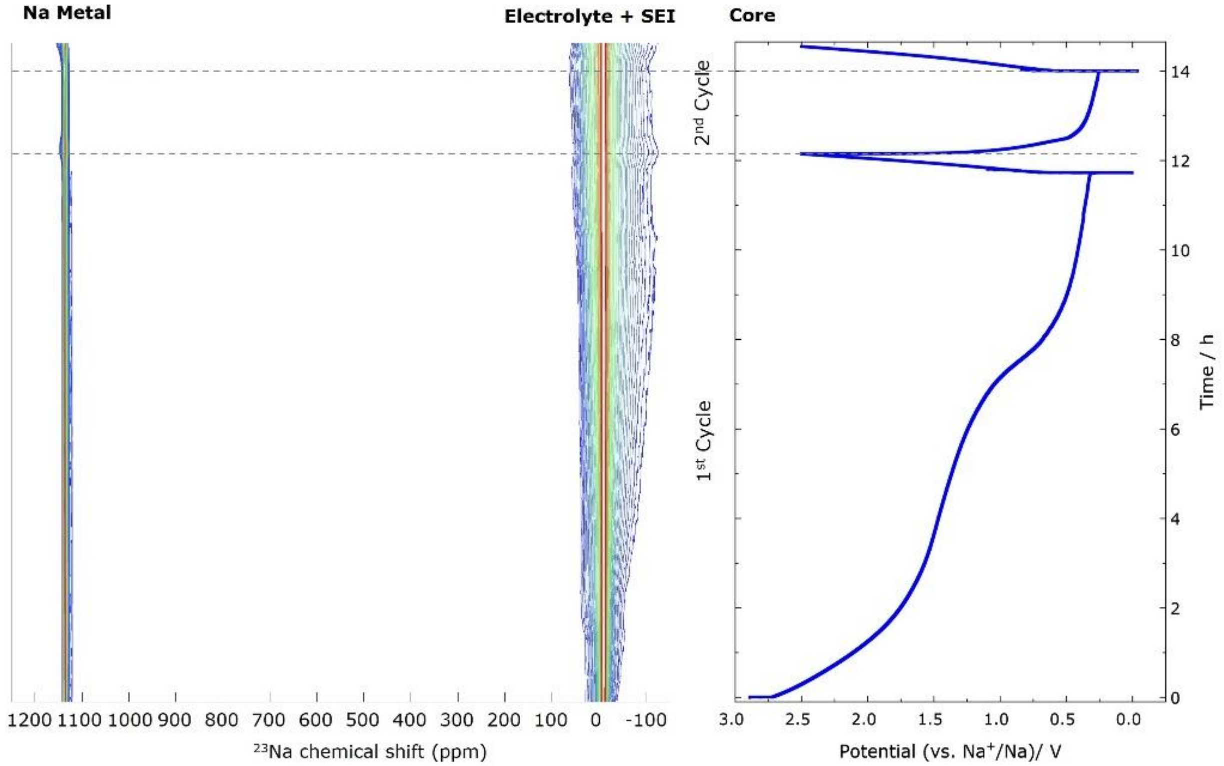

Figure S7. Operando ^{23}Na solid-state NMR spectra of an electrochemical half-cell comprising the core material, sodium metal, and $NaPF_6$ electrolyte. The corresponding electrochemistry for the 1st and 2nd cycles is shown on the right-hand side of the spectrum.

Calculations for Porosity of Particles, Shell Thickness and Dynamic Vapour Sorption Surface Areas

Calculation 1: Porosity

Assumptions: initial total pore volume per g Core V_{pores} = 1.18 cm^3g^{-1}, 30 wt% carbon added; outer volume unchanged.

Case 1: Density estimated to be ρ = 1.8 g cm^{-3}

Solid volume per Core

$$V_{solid,0} = \frac{1}{\rho} = \frac{1}{1.8\ g\ cm^{-3}} = 5.556 \times 10^{-1}\ cm^3 g^{-1}$$

Total Volume per g Core

$$V_{tot,0} = V_{solid,0} + V_{pores,0} = 5.556\ \times 10^{-1}\ cm^3 g^{-1} + 1.18\ cm^3 g^{-1} = 1.7356\ cm^3 g^{-1}$$

Initial porosity (p_0)

$$p_0 = \frac{V_{pores,0}}{V_{tot,0}} = \frac{1.18}{1.7356} \times 100 = 67.99\ \%$$

Added solid volume (absolute) per 1 g Core

$$V_{add} = \frac{0.30\ g}{\rho} = \frac{0.30\ g}{1.8\ g\ cm^{-3}} = 1.6667\ \times 10^{-1} cm^3$$

Remaining pore volume assuming pore filling (absolute) per 1 g Core

$$V_{pores,new} = V_{pores,0} - V_{add} = 1.18\ cm^3 - 1.6667\ \times 10^{-1} cm^3 = 1.0133\ cm^3$$

Porosity after addition (p_{new}) assuming pore filling

$$p_{new} = \frac{V_{pores,new}}{V_{tot,0}} = \frac{1.0133\ cm^3}{1.7356\ cm^3} \times 100 = 58.39\%$$

TPV (per g composite) assuming pore filling

$$TPV_{comp} = \frac{V_{pores,new}}{m_{composite}} = \frac{1.0133\ cm^3}{1.3\ g} = 0.779\ cm^3 g^{-1}$$

Case 2: Density estimated to be ρ = 2.1 g cm^{-3}

Solid volume per Core

$$V_{solid,0} = \frac{1}{\rho} = \frac{1}{2.1\ g\ cm^{-3}} = 4.7619 \times 10^{-1}\ cm^3 g^{-1}$$

Total Volume per g Core

$$V_{tot,0} = V_{solid,0} + V_{pores,0} = 4.7619\ \times 10^{-1}\ cm^3\ g^{-1} + 1.18\ cm^3\ g^{-1} = 1.6562\ cm^3 g^{-1}$$

Initial porosity (p_0)

$$p_0 = \frac{V_{pores,0}}{V_{tot,0}} = \frac{1.18\ cm^3\ g^{-1}}{1.6562\ cm^3\ g^{-1}} \times 100 = 71.25\ \%$$

Added solid volume (absolute) per 1 g Core

$$V_{add} = \frac{0.30\ g}{p} = \frac{0.30\ g}{2.1\ g\ cm^{-3}} = 1.4286\ \times 10^{-1} cm^3$$

Remaining pore volume (absolute) per 1 g Core

$$V_{pores,new} = V_{pores,0} - V_{add} = 1.18\ cm^3 - 1.4286\ \times 10^{-1} cm^3 = 1.0371\ cm^3$$

Porosity after addition (p_{new}) assuming pore filling

$$p_{new} = \frac{V_{pores,new}}{V_{tot,0}} = \frac{1.0371\ cm^3}{1.6562\ cm^3} \times 100 = 62.62\%$$

TPV (per g composite) assuming pore filling

$$TPV_{comp} = \frac{V_{pores,new}}{m_{composite}} = \frac{1.0371}{1.3\ g} = 0.798\ cm^3 g^{-1}$$

Calculation 2: Reverse Case (Mass needed for target TPV = 0.01 $cm^3\ g^{-1}$)

Required mass (ρ = 1.8 g cm^{-3})

$$m_{add} = \rho\left(V_{pores,0} - V_{target}\right) = 1.8\ g\ cm^{-3}\ \times (1.18\ cm^3\ g^{-1} - 0.01\ cm^3\ g^{-1}) = 2.1060\ g\ g^{-1} \approx 211\ \%$$

Required mass (ρ = 2.1 g cm^{-3})

$$m_{add} = \rho\left(V_{pores,0} - V_{target}\right) = \ 2.1\ g\ cm^{-3}\ \times (1.18\ cm^3\ g^{-1} - 0.01\ cm^3\ g^{-1}) = 2.4570\ g\ g^{-1} \approx 246\ \%$$

Calculation 3: Coating Thickness Calculations

We treat spherical particles and increase the mass by 30% ($m_{core} = 0.77 \cdot m_{core\text{-}shell}$); to illustrate the geometric steps:

Case 3.1 Skeletal carbon density ρ = 1.8 g cm^{-3}

Effective density Core (volume from calculation 1):

$$\rho_{core} = \frac{mass}{volume} = \frac{1\ g}{1.7356\ cm^3} = 0.576\ g\ cm^{-3}$$

Effective density Core-Shell assuming shell formation:

$$V_{core-shell} = \ V_{core} + \ V_{shell} = \ 1.7356\ cm^3 + \frac{0.3\ g}{1.8\ g\ cm^{-3}} = 1.903\ cm^3$$

$$\rho_{core-shell} = \frac{mass_{core-shell}}{V_{core-shell}} = \ \frac{1.3\ g}{1.903\ cm^3} = 0.683\ gcm^{-3}$$

Area of N spherical particles ≈ measured SSA:

$$A = N\ \times\ 4\pi R^2$$

$$N = \frac{A}{4\pi R^2}$$

Mass of N spherical particles:

$$\begin{aligned} m &= \rho \times V \\ &= \rho \times N \times \frac{4}{3}\pi R^3 \\ &= \rho \times \frac{A}{4\pi R^2} \times \frac{4}{3}\pi R^3 \\ &= \frac{\rho \times A \times R}{3} \end{aligned}$$

$$R = \frac{3m}{\rho A} = \frac{3}{\rho \times SSA}$$

Radius of core-shell particle:

$$R_{core-shell} = \frac{3\ m\ (mass\ of\ sample)}{area\ (overall\ surface\ area) \times density\ (eff\ coated\ particle)} = \frac{3\ (1\ g)}{14m^2\ \times 0.683\ gcm^{-3}}$$
$$= 313.7\ nm$$

Volume of core-shell particle:

$$V_{core-shell} = \frac{4}{3}\pi R^3_{core-shell} = \frac{4}{3}\pi(3.137\ \times 10^{-7}\ cm)^3 = 1.294\ \times 10^{-13} cm^3$$

Mass of core-shell particle:

$$m_{core-shell} = V_{core-shell}\rho_{core-shell} = 1.294\ \times 10^{-13} cm^3 \times 0.683\ g\ cm^{-3} = 8.835\ \times 10^{-14} g$$

Mass of core particle:

$$m_{core} = 0.77\ m_{core-shell} = 0.77 \times 8.835 \times 10^{-14} g = 6.803 \times 10^{-14} g$$

Volume of core particle:

$$V_{core} = \frac{m_{core}}{p_{core}} = \frac{6.803 \times 10^{-14} g}{0.576\ g\ cm^{-3}} = 1.181 \times 10^{-13}\ cm^3$$

Radius of core particle:

$$R_{\ core} = \left(\frac{3V_1}{4\pi}\right)^{1/3} = \left(\frac{3 \times 1.181 \times 10^{-13}\ cm^3}{4\pi}\right)^{1/3} = 3.044\ \times 10^{-5}\ cm$$

Thickness of Shell:

$$\Delta R = R_{core-shell} - R_{core} = 313.7\,nm - 304.4\,nm = 9.3\,nm$$

Simplified shell thickness assuming negligible curvature (i.e. flat surface without assumed shape, valid for spheres if shell thickness << core thickness):

$$thickness = \frac{Volume\ of\ coating}{Area\ of\ coating} = \frac{\left(\frac{mass}{density}\right)}{area} = \frac{\left(\frac{0.23\ g}{1.8\ gcm^{-3}}\right)}{(1.4\ \times 10^5\ cm^2)} = 9.1\ nm$$

Case 3.2 Skeletal carbon density ρ = 2.1g cm^{-3}

Effective density core (volume from calculation 1):

$$\rho_{core} = \frac{mass}{volume} = \frac{1\ g}{1.6562\ cm^3} = 0.6038\ g\ cm^{-3}$$

Effective density Core-Shell assuming shell formation:

$$V_{core-shell} = V_{core} + V_{shell} = 1.6562\ cm^3 + \frac{0.3\ g}{2.1\ gcm^{-3}} = 1.799\ cm^3$$

$$p_{core-shell} = \frac{mass_{core-shell}}{volume_{core-shell}} = \frac{1.3\ g}{1.799\ cm^3} = 0.722\ gcm^{-3}$$

Area of N spherical particles ≈ measured SSA:

$$A = N \times 4\pi R^2$$
$$N = \frac{A}{4\pi R^2}$$

Mass of N spherical particles:

$$\begin{aligned} m &= \rho \times V \\ &= \rho \times N \times \frac{4}{3}\pi R^3 \\ &= \rho \times \frac{A}{4\pi R^2} \times \frac{4}{3}\pi R^3 \\ &= \frac{\rho \times A \times R}{3} \end{aligned}$$

$$R = \frac{3m}{\rho A} = \frac{3}{\rho \times SSA}$$

Radius of core-shell:

$$R_{core-shell} = \frac{3\ m\ (mass\ of\ sample)}{area\ (overall\ surface\ area) \times density\ (eff\ coated\ particle)} = \frac{3\ (1.000\ g)}{14\ m^2\ \times 0.722\ g\ cm^{-3}} = 296.8\ nm$$

Volume of core-shell:

$$V_{core-shell} = \frac{4}{3}\pi R_{core-shell}^3 = \frac{4}{3}\pi(296.8\ \times 10^{-7}\ cm)^3 = 1.095\ \times 10^{-13} cm^3$$

Mass of core-shell:

$$m_{core-shell} = V_{core-shell}p_{core-shell} = 1.095 \times 10^{-13}cm^3 \times 0.722\ gcm^{-3} = 7.907 \times 10^{-14}g$$

Mass of core:

$$m_{core} = 0.77\ m_{core-shell} = 0.77 \times 7.907 \times 10^{-14}g = 6.088 \times 10^{-14}g$$

Volume of core:

$$V_{core} = \frac{m_{core}}{p_{core}} = \frac{6.088 \times 10^{-14}g}{0.6038\ g\ cm^{-3}} = 1.008 \times 10^{-13}\ cm^3$$

Radius of core:

$$R_{core} = \left(\frac{3V_1}{4\pi}\right)^{1/3} = \left(\frac{3 \times 1.181 \times 10^{-13}\ cm^3}{4\pi}\right)^{1/3} = 2.887 \times 10^{-5}\ cm$$

Thickness of Shell:

$$\Delta R = R_{core-shell} - R_{core} = 296.8\ nm - 288.7\ nm = 8.1\ nm$$

Simplified shell thickness assuming negligible curvature (i.e. flat surface without assumed shape, valid for spheres if shell thickness << core thickness):

$$thickness = \frac{Volume\ of\ coating}{Area\ of\ coating} = \frac{\left(\frac{mass}{density}\right)}{area} = \frac{\left(\frac{0.23\ g}{2.1\ gcm^{-3}}\right)}{(1.4 \times 10^5\ cm^2)} = 7.8\ nm$$

Calculation 4: DEC Vapor Sorption

To obtain the effective surface area of DEC, we performed Grand Canonical Monte Carlo simulation at 298.15 K and 0.05 ≤ p/p_0 ≤ 0.95. We determined the effective surface area A_{DEC} from the number of molecules adsorbed averaged over 100,000 Monte Carlo steps:

$$A_{\mathrm{DEC}} = \frac{A_{\mathrm{unit\ cell}}}{0.5 \times N_{\mathrm{adsorbed}}}$$

where $A_{\mathrm{unit\ cell}} = 11.81\ \mathrm{nm}^2$ is the area of the graphene supercell and N_{adsorbed} is the average number of DEC adsorbed in this unit cell. The factor of 0.5 accounts for the double-sided adsorption on a graphene monolayer. The simulations revealed that a full monolayer of DEC could be adsorbed already at p/p_0 = 0.05 (Fig. S8b), and increasing the pressure led to slightly higher loading (Fig. S8a) due to second-layer adsorbates or molecular re-orientation into vacuum (Fig. S8c). At p/p_0 = 0.05 where such effects are

relatively suppressed, we obtained an average loading of $(38.9 \pm 0.5 \frac{\text{molecules}}{\text{unit cell}})$, giving an effective surface area of (0.607 ± 0.008) nm^2 per DEC molecule.

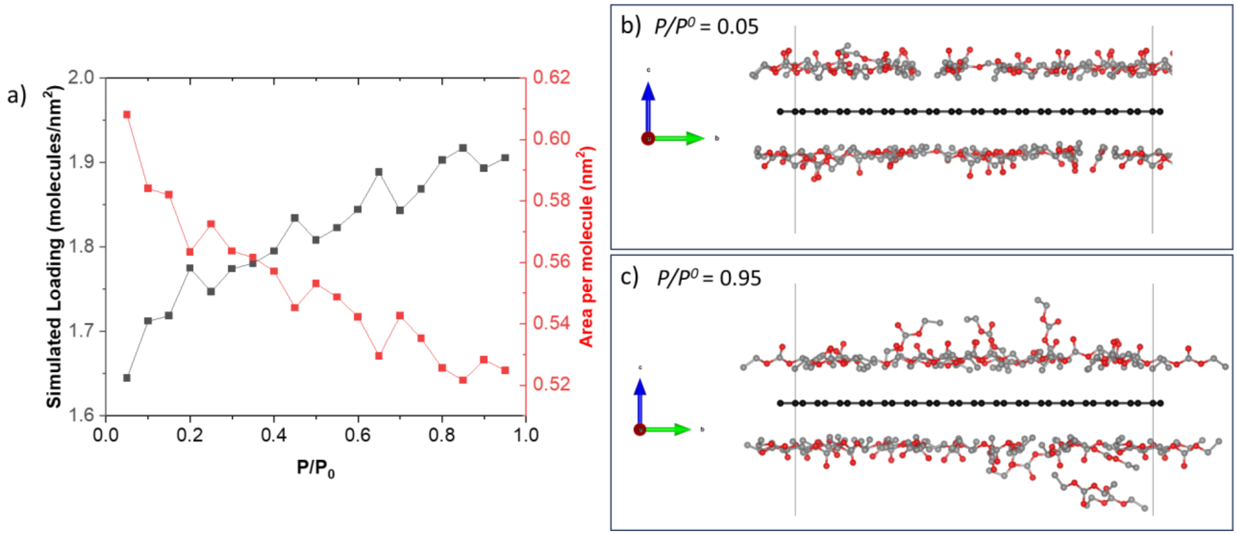

Figure S8. a) Plot of GCMC-simulated loading of DEC adsorption per unit area at various pressures. (b,c) Representative adsorption structure of DEC (b) with monolayer-like structure (p/p_0 = 0.05, 39 molecules per unit cell) and (c) with partial second layer adsorption (P/P^0 = 0.95, 44 molecules per unit cell).

The experimentally derived specific surface area (SSA) measured with DEC vapor sorption was calculated by converting the adsorbed mass of DEC into the number of molecules and then multiplying by the effective surface area of DEC. The adsorbed DEC mass (m_{ads}) was converted into number of molecules using the molar mass of DEC (M = 118.13 g mol^{-1}) and the Avogadro's constant (N_A = 6.022 × 10^{23} mol^{-1}):

$$N = \frac{m_{ads} \times N_A}{M}$$

The overall surface area covered by these molecules was then obtained from the Monte-Carlo predicted effective surface area of a single DEC molecule on graphene (A_{DEC} = 0.61 nm^2):

$$A_{tot} = N \times A_{\mathrm{DEC}}$$

Finally, the specific surface area was determined by normalizing the total area to the sample mass (m_s):

$$SSA = \frac{A_{tot}}{m_s}$$

Using this procedure, the pristine Core with an adsorbed DEC mass of 13.25 mg yields an SSA of 1896 m^2 g^{-1}, whereas the coated Core–Shell with 0.21 mg DEC uptake corresponds to only 13 m^2 g^{-1}. These results confirm that the CVD treatment drastically reduces the solvent-accessible surface area by almost two orders of magnitude. It is worth mentioning that these numbers in the same order of magnitude as the BET surface area derived from N_2 sorption, and deviations could be attributed to differences in pore accessibility of the molecules.
